# Differential Expression of Glycolysis-Related Proteins in Follicular Neoplasms versus Hürthle Cell Neoplasms: A Retrospective Analysis

**DOI:** 10.1155/2017/6230294

**Published:** 2017-07-16

**Authors:** Hye Min Kim, Ja Seung Koo

**Affiliations:** Department of Pathology, Yonsei University College of Medicine, Seoul, Republic of Korea

## Abstract

**Purpose:**

Although currently classified as variants of follicular neoplasms (FNs), Hürthle cell neoplasms (HCNs) exhibit distinct biological characteristics. Hence, the metabolism of both neoplasms may also be different. The aims of this study were to investigate and compare the expression of glycolysis-related proteins in HCNs and FNs and to determine the clinical implications of such expression.

**Methods:**

Tissue microarrays were constructed with 265 samples of FNs (112 follicular carcinomas (FCs) and 153 follicular adenomas (FAs)) as well as 108 samples of HCNs (27 Hürthle cell carcinomas (HCCs) and 81 Hürthle cell adenomas (HCAs)). Immunohistochemical staining for the glycolysis-related molecules Glut-1, hexokinase II, CAIX, and MCT4 was performed.

**Results:**

The expression levels of Glut-1, hexokinase II, CAIX, and MCT4 were significantly higher in HCNs than in FNs (*p* < 0.001). Glut-1, hexokinase II, CAIX, and MCT4 expression levels were highest in HCC, followed by HCA, FC, and FA (all *p* < 0.001). In HCC, hexokinase II positivity was associated with large tumor size (>4 cm) (*p* = 0.046), CAIX positivity with vascular invasion (*p* = 0.005), and MCT4 positivity with extrathyroidal extension (*p* = 0.030).

**Conclusion:**

The expression levels of the glycolysis-related proteins Glut-1, hexokinase II, CAIX, and MCT4 were higher in HCNs than in FNs and in HCCs than in HCAs.

## 1. Introduction

The metabolism of malignant tumors is characterized by the Warburg effect, in which a metabolic shift from oxidative phosphorylation in the mitochondria towards glycolysis occurs in tumor cells [1]. Key molecules involved in regulating glycolysis and its products include Glut-1, hexokinase II, CAIX, and MCT4. Glut-1 is a glucose transporter [2] while hexokinase II is the initiating enzyme in glycolysis that phosphorylates glucose to produce glucose-6-phosphate [3]. CAIX leads to the reversible hydration of carbon dioxide to neutralize the acidification caused by lactate formed during glycolysis [4], and the MCT4 channel exports lactate produced by glycolysis out of the cell [5].

Currently, the World Health Organization classifies Hürthle cell neoplasm (HCN) as an oncocytic variant of follicular neoplasm (FN) [6]. Hürthle cells are considered an oxyphilic variant of follicular epithelial cells and are characterized by large-sized cells with polygonal to square shapes, distinct borders, hyperchromatic nuclei with prominent nucleoli, and abundant eosinophilic granular cytoplasm owing to the accumulation of large numbers of intracytoplasmic mitochondria. Hürthle cell adenomas (HCAs) account for 10–15% of follicular adenomas (FAs), while Hürthle cell carcinomas (HCCs) comprise 20–25% of follicular carcinomas (FCs) [7]; however, several studies have suggested that HCN could be a distinct disease. The rate of malignancy in FNs ranges from 15% to 30%, compared to a higher rate of 25–45% in HCNs [8–12]. Compared to FC, HCC is prone to metastasize to the lymph nodes, soft tissues of the neck, [13], and distant sites [14] and exhibits a higher rate of resistance to iodine therapy. Therefore, the prognosis of HCC is poor; this disease carries a higher rate of recurrence and mortality [14–17]. Furthermore, genomic analysis of HCCs suggests that it may represent a unique class of thyroid malignancies [18], and molecular studies revealed that the *TERT C228T* promoter mutation is common in HCNs [19].

While HCN and FN tumors exhibit different biological characteristics, a previous study using [^18^F]-2-fluoro-2-deoxy-D-glucose (^18^F-FDG) positron emission tomography (PET) also showed that HCAs exhibit higher focal ^18^F-FDG uptakes and maximum standardized update values (SUVmax) than FAs [20]. Therefore, it could be speculated that the metabolic features of HCN and FN differ; however, this has not been researched extensively to date. In a previous study, we have demonstrated differential expression of glycolysis-related protein among different types of thyroid cancer [21]. Therefore, the aims of this study were to investigate the expression of glycolysis-related proteins in HCN and FN and to examine the clinical implications of any differences in expression levels.

## 2. Materials and Methods

### 2.1. Patient Selection

Patients who were diagnosed with FN and HCN after surgery at the Severance Hospital between January 2000 and December 2013 and with available paraffin blocks and slides for histologic evaluation were included in this study. All cases were retrospectively reviewed by a thyroid pathologist (JSK), and histological evaluation was performed after hematoxylin and eosin staining. Clinicopathologic data were obtained from the patients' medical records and included age at diagnosis, disease recurrence, metastasis, current status, and duration of follow-up. The tumor size, location (right or left lobe), extent (confined to the thyroid parenchyma or with extrathyroidal spread), and number of metastatic lymph nodes were also noted after reviewing the slides and surgical pathology reports. This study was approved by the Institutional Review Board of Yonsei University Severance Hospital.

### 2.2. Tissue Microarray

Representative areas were selected on hematoxylin and eosin-stained slides, and a corresponding spot was marked on the surface of the matching paraffin block. Three-millimeter-sized tissue cores were extracted by using a manual tissue arrayer from the selected areas and placed into a 6 × 5 recipient block. More than two tissue cores were extracted from each sample to minimize extraction bias. Each tissue core was assigned a unique tissue microarray location number that was linked to a database containing other clinicopathologic data.

### 2.3. Immunohistochemistry

Antibodies used for immunohistochemistry are listed in Table 1. All immunohistochemistry was performed with formalin-fixed, paraffin-embedded tissue sections using an automatic immunohistochemistry staining device (Benchmark XT, Ventana Medical System, Tucson, AZ, USA). Briefly, 5 *μ*m-thick formaldehyde-fixed paraffin-embedded tissue sections were transferred onto adhesive slides and dried at 62°C for 30 minutes. Standard heat epitope retrieval was performed for 30 minutes in ethylene diamine tetraacetic acid, pH 8.0, in the autostainer. The samples were then incubated with primary antibodies. Afterwards, the sections were incubated with biotinylated anti-mouse immunoglobulins, peroxidase-labeled streptavidin (LSAB Kit, DakoCytomation), and 3,30-diaminobenzidine. Negative control samples were processed without the primary antibody. Positive control tissues were used as per the manufacturer's recommendation. Slides were counterstained with Harris hematoxylin. Optimal primary antibody incubation times and concentrations were determined by serial dilutions of each immunohistochemical assay using a tissue block fixed and embedded exactly as performed for the samples.

### 2.4. Interpretation of Immunohistochemical Staining

Immunohistochemical markers were accessed by light microscopy. The stained slides were semiquantitatively evaluated as previously described [22]. Tumor cell staining was assessed as 0: negative or weak immunostaining in <1% of the tumor cells, 1: focal expression in 1–10% of tumor cells, 2: positive in 11–50% of tumor cells, and 3: positive in 51–100% of tumor cells. These evaluations were applied over the entire area of the tumor, which was scored as follows; 0-1: negative, 2: low-positive, and 3: high-positive. For the Ki-67 labeling index (Ki-67 L.I.), grading was performed as previously described, with some modifications [23, 24]. Tumor cells with Ki-67 L.I. < 3% was graded as 0, 3% ≤ Ki-67 L.I. ≤ 5% as 1, and 5% < Ki-67 L.I. as 2. “Glycolysis type” samples were defined as those positive for two or more of the markers Glut-1, hexokinase II, CAIX, and MCT4; otherwise, the sample was defined as nonglycolysis type.

### 2.5. Statistical Analysis

Data were analyzed using IBM SPSS Statistics for Windows, Version 21.0 (IBM Corp. Released 2012, Armonk, NY, USA). For determination of statistical significance, the Student's *t*-test and Fisher's exact test were used for continuous and categorical variables, respectively. For analyzing data with multiple comparisons, a corrected *p* value with the application of the Bonferroni multiple comparison procedure was used. Correlation among glycolysis-related proteins, clinicopathologic factors, and Ki-67 L.I. was compared using the Spearman's rho. Statistical significance was set to *p* < 0.05. Cox proportional hazards model with univariate and multivariate analyses was used to evaluate the prognostic factors for disease-free and overall survival.

## 3. Results

### 3.1. Basal Characteristic of Follicular Neoplasms and Hürthle Cell Neoplasms

This study included 265 patients with FN: 153 with FA and 112 with FC. Of the 112 FCs, 99 cases were of the minimally invasive type and 13 were widely invasive. Clinicopathologic features of the FCs are presented in Supplementary Table 1 available online at https://doi.org/10.1155/2017/6230294. Additionally, 108 patients with HCN were included, 81 with HCA and 27 with HCC. The clinicopathologic features of HCN are presented in Supplementary Table 1.

### 3.2. Expression of Glycolysis-Related Proteins in Follicular Neoplasms and Hürthle Cell Neoplasms

**Figure 1:**
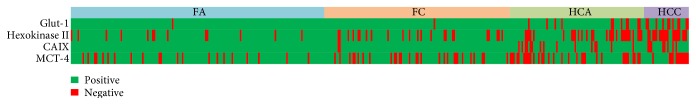
Heat map of glycolysis-related proteins found in follicular neoplasms and Hürthle cell neoplasms. (FA: follicular adenoma; FC: follicular carcinoma; HCA: Hürthle cell adenoma; HCC: Hürthle cell carcinoma).

**Figure 2:**
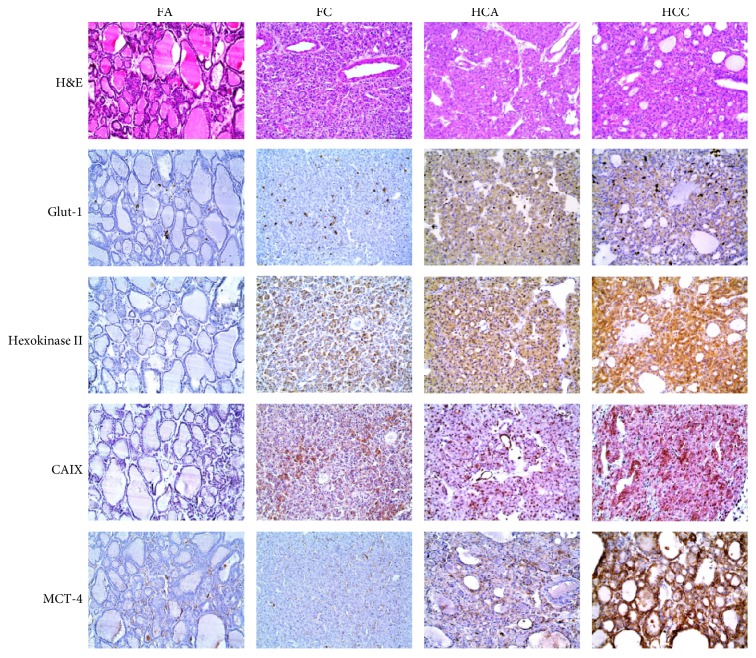
Expression of glycolysis-related proteins in follicular neoplasms and Hürthle cell neoplasms. The expression of Glut-1, hexokinase II, CAIX, and MCT4 was significantly higher in Hürthle cell neoplasms compared to follicular neoplasms and in Hürthle cell carcinomas than in Hürthle cell adenomas. (FA: follicular adenoma; FC: follicular carcinoma; HCA: Hürthle cell adenoma; HCC: Hürthle cell carcinoma).

**Figure 3:**
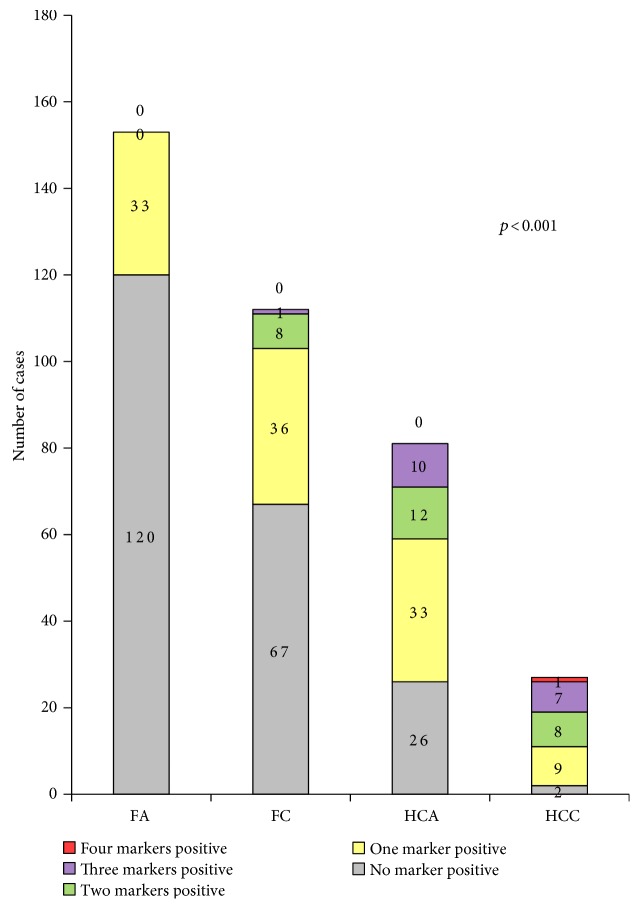
Number of positive glycolytic markers in follicular neoplasms and Hürthle cell neoplasms. (FA: follicular adenoma; FC: follicular carcinoma; HCA: Hürthle cell adenoma; HCC: Hürthle cell carcinoma).

**Figure 4:**
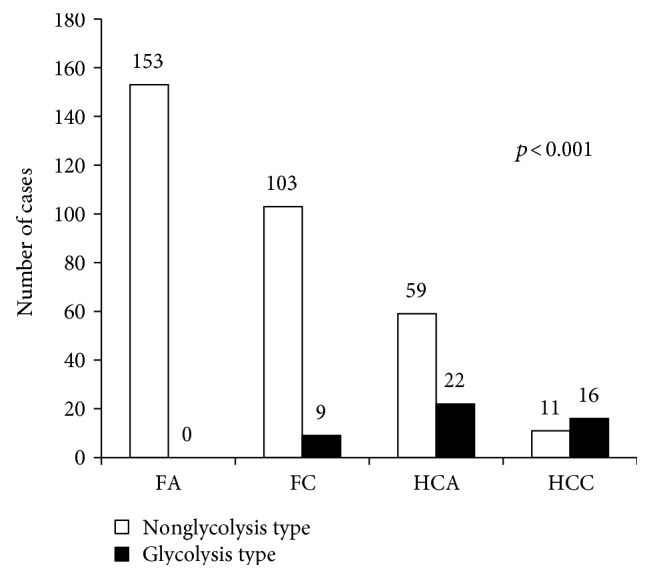
Glycolysis type in follicular neoplasms and Hürthle cell neoplasms. (FA: follicular adenoma; FC: follicular carcinoma; HCA: Hürthle cell adenoma; HCC: Hürthle cell carcinoma).

The expression of Glut-1, hexokinase II, CAIX, and MCT4 was significantly higher in HCNs than in FNs (*p* < 0.001, Table 2 and Figure 1). When comparing the expression of glycolysis-related proteins between FA, FC, HCA, and HCC, Glut-1, hexokinase II, CAIX, and MCT4 expression was highest in HCC, followed by HCA, FC, and FA (all *p* < 0.001, Table 3 and Figure 2). Furthermore, the number of positive markers among the four glycolysis-related markers was examined in FA, FC, HCA, and HCC. The percentage of cases with the number of positive markers was the highest in HCC, followed by HCA, FC, and FA (Table 4 and Figure 3). The ratio of glycolysis type was the highest in HCC, followed by HCA, FC, and FA (Figure 4).

### 3.3. Correlation between the Expression of Glycolysis-Related Proteins and Clinicopathologic Factors in Hürthle Cell Carcinoma

**Figure 5:**
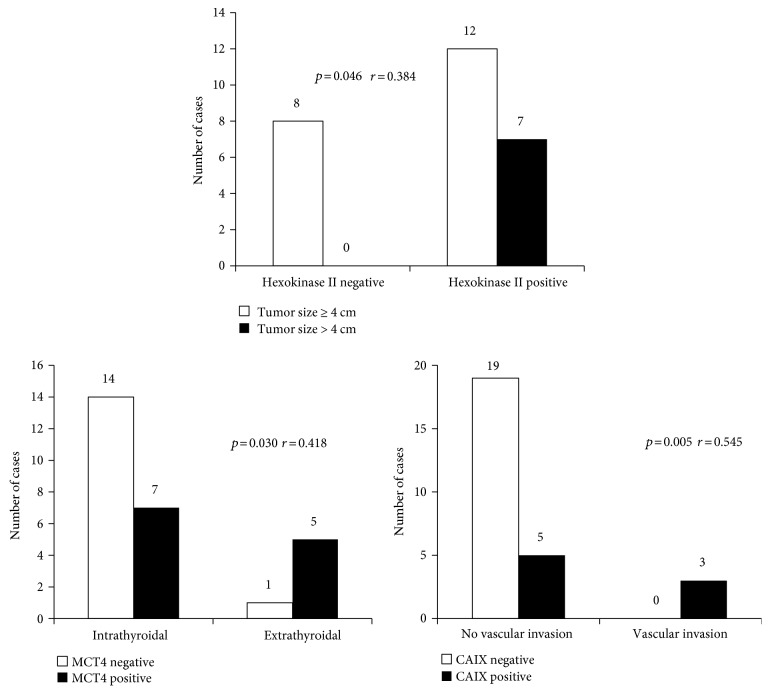
Correlation between the expression of glycolysis-related proteins and clinicopathologic factors in Hürthle cell carcinoma.

We investigated the correlation between the expression of glycolysis-related proteins and clinicopathologic factors in HCC. Hexokinase II positivity was associated with large tumor size (>4 cm) (*r* = 0.384, *p* = 0.046), CAIX positivity with vascular invasion (*r* = 0.545, *p* = 0.005), and MCT4 positivity with extrathyroidal extension (*r* = 0.418, *p* = 0.030) (Figure 5).

### 3.4. Correlation between the Expression of Glycolysis-Related Proteins and Ki-67 Labeling Index in Follicular Carcinoma and Hürthle Cell Carcinoma

Next, we evaluated the correlation between Ki-67 L.I. and the expression of glycolysis-related proteins in FC and HCC. Correlation analysis showed that Ki-67 L.I. was related to MCT4 in FC (*r* = 0.187, *p* = 0.048) and GLUT1 expression (*r* = 0.419, *p* = 0.029) in HCC (Table 5).

### 3.5. The Impact of the Expression of Glycolysis-Related Proteins on Prognosis

Finally, we investigated the impact of the clinical parameters and the expression of glycolysis-related proteins in the clinical outcomes of patients with FC and HCC. During the follow-up period, 11 patients experience disease recurrence, while 5 patients had suffered death. In multivariate analysis, female sex (OR 0.201, 95% CI 0.061-0.060, *p* = 0.008) and extrathyroidal extension (OR 4.937, 95% CI 1.503–16.208, *p* = 0.008) was related to disease-free survival (Table 6), while only lymph node metastasis (OR 15.742, 95% CI 1.723–143.770, *p* = 0.014) was related to overall survival (Table 7). However, the expression of glycolysis-related proteins Glut-1, hexokinase II, CAIX, and MCT4 levels was not significantly related to disease-free and overall survival (Tables 6 and 7).

## 4. Discussion

We found that the expression levels of the glycolysis-related proteins Glut-1, hexokinase II, CAIX, and MCT4 were significantly higher in HCNs than in FNs. FDG is a glucose analog, and ^18^F-FDG is the most commonly used radiotracer in PET/computed tomography image acquisition. As glucose metabolism is increased in glycolytic tissues, such as those with malignancies, such tissues increase their uptake of ^18^F-FDG. Our findings are consistent with those of a previous study showing that HCAs exhibit a higher focal FDG uptake and higher SUVmax compared to FA, suggesting that glycolysis is more active in HCAs [20, 25].

There is ample evidence of the differential expression of glycolysis-related proteins in tumors. In a genomic dissection study, the PI3K-AkT-mTOR and Wnt/*β*-catenin pathways were shown to be activated in HCC, which exhibited a different molecular profile than FN [18]. The PI3K-Akt pathway plays a pivotal role in translocating Glut-1 from the cytoplasm to the plasma membrane in cells of endocrine organs such as the thyroid gland [26] and pancreas [27]. Furthermore, hexokinase II was shown to play a critical role in the proliferation of hepatocellular carcinoma cells in an Akt signaling pathway-dependent manner [28]. Lee et al. demonstrated that Wnt signaling activation in breast cancer cells promotes glycolysis, which is indirectly mediated by Snail, the transcriptional repressor of cytochrome c oxidase [29]. Therefore, it makes sense that the levels of glycolysis-related proteins are increased in HCC, since PI3K-AkT-mTOR and Wnt/*β*-catenin pathways are activated in these tumors. Additionally, all HCNs express the hTERT protein [30] and *TERT C228T* promoter mutation is common in HCNs [19]. HCCs also exhibit markedly shortened telomeres [19, 30]. In previous studies, several genes involved in the glycolytic pathway were shown to be downregulated following *TERT* knockdown, suggesting that TERT directly regulates cancer cell metabolism, especially glycolysis [31, 32]. These findings suggest that the higher expression of glycolysis-related proteins in HCNs is associated with TERT activity; however, additional studies are required to clarify this.

Glycolytic marker positivity was the highest in HCCs, followed by HCAs, FCs, and FAs. The proportions of “glycolysis type” samples followed the same pattern. The expression levels of glycolysis-related proteins were higher in carcinomas than in adenomas in both HCNs and FNs, suggesting that cancer cells utilize glycolysis for proliferation and cell growth. In a previous study, autophagy and senescence in cancer-associated fibroblasts were shown to contribute to tumor growth and metastasis via glycolysis and ketone production [33]. Furthermore, glycolysis triggered tumor metastasis by promoting resistance against anoikis, which is a barrier to tumor metastasis, in mammary epithelial cell lines [34], whereas inhibition of glycolysis in head and neck squamous cell carcinoma suppressed tumor growth and metastasis [35]. These findings are consistent with our results that showed that the expression of glycolysis-related proteins is higher in carcinomas than in adenomas; however, the underlying mechanisms of increased expression of glycolysis-related proteins in HCC require further investigation.

We have demonstrated that hexokinase II positivity was associated with large tumor size and CAIX positivity with vascular invasion, whereas MCT4 positivity was linked to extrathyroidal extension. Although there are no previous studies directly comparing glycolysis activities between FN and HCN, a previous study did evaluate glycolysis-related protein expression in breast solid papillary carcinoma, with results similar to ours [36]. Separately, the efflux of hydrogen ions mediated by CAIX neutralizes the intracellular acidic microenvironment that can cause the degradation of the extracellular matrix and basement membrane [37]. Therefore, it can be speculated that higher expression of CAIX influences the invasive growth of tumor cells. Previously, stromal MCT4 expression was shown to reflect the oxidative stress experienced by cancer-associated fibroblasts [38]. Hence, it is possible that oxidative stress is increased in cancer-associated fibroblasts in the event of extrathyroidal extension following the aggressive growth of cancer cells, resulting in increased expression of MCT4. Likewise, Ki-67, proliferation marker that is expressed in tumor cells also correlated with MCT4 in FC. However, this hypothesis should be tested in future studies. In a previous study that evaluated glycolysis-related protein expression in colorectal carcinoma, tumor size was associated with hexokinase II positivity; this makes sense because the glycolysis pathway provides energy to cancer cells and serves as a biomoleculer precursor of proliferation [39]. Additionally, hexokinase II hampers cancer cell apoptosis and increases mitochondrial stability and cell proliferation activity via PI3K-dependent and PI3K-independent pathways in different types of carcinomas [28, 40–43]; these data are consistent with our results.

The clinical significance of this study is that glycolysis-related proteins could be a potential therapeutic target in the treatment of HCC. As lymph node metastasis in HCC reportedly occurs in 5.3–13% of cases [19, 44–46], aggressive surgical treatment or complete thyroidectomy is necessary [47]. Several adjuvant therapy treatment options have been suggested following surgical resection, including thyroxine suppression, radioiodine, cervical radiation, directed therapy for distant metastasis, and systemic therapy [47]. However, the optimal treatment option is still unclear. In previous studies, the targeting of Glut-1 [27, 48–50], hexokinase II [51, 52], CAIX [53, 54], and MCT4 [55] suppressed tumor growth, invasion, and metastasis. Therefore, targeting glycolysis-related proteins in HCC might also be a promising therapy; however, this remains to be verified in future studies that include clinical trials.

There are several limitations in this study. First, we used immunohistochemistry to evaluate glycolysis-related protein expression in FN and HCN, which has a limitation in quantification and with a possibility of interobserver bias. In addition, the results of immunohistochemical staining could be different depending on antibody reactivity. Second, as we used TMA, not whole section of the tumor specimen to evaluate the histological examination, there could be an extraction bias in TMA construction. However, as TMA has shown to be a reliable method for immunohistochemical analysis in clinicopathological characterization in various tissues including thyroid gland, using TMA to evaluate glycolysis-related protein expression might also be applicable. Third, although glycolysis-related protein expression was higher in HCNs and carcinomas compared to FNs and adenomas, the prognostic implication regarding disease-free and overall survival was not significant. Further investigation is required to elucidate the role of glycolysis-related protein expression in predicting patient prognosis.

## 5. Conclusion

The expression levels of the glycolysis-related proteins Glut-1, hexokinase II, CAIX, and MCT4 are higher in HCNs compared to FNs and higher in HCCs than in HCAs. Moreover, our results showed that overexpression of each of these markers is associated with more aggressive tumor characteristics, thus providing potential therapeutic targets for HCN and FC.

## Supplementary Material

Supplementary Table 1. Basal characteristics of follicular carcinoma and Hürthle cell neoplasm.

## Figures and Tables

**Table 1:** Source, clone, and dilution of antibodies used in this study.

Antibody	Clone	Dilution	Company
Glut-1	SPM498	1 : 200	Abcam, Cambridge, UK
Hexokinase II	3D3	1 : 200	Abcam, Cambridge, UK
CAIX	Polyclonal	1 : 100	Abcam, Cambridge, UK
MCT4	Polyclonal	1 : 100	Santa Cruz, CA, USA
Ki-67	MIB-1	1 : 150	Dako, Denmark AS, Glostrup, Denmark

**Table 2:** Expression of glycolysis-related proteins in follicular neoplasms and Hürthle cell neoplasms.

Parameters	Total *N* = 373 (%)	Follicular neoplasm *n* = 265 (%)	Hürthle cell neoplasm *n* = 108 (%)	*p* value
GLUT-1				<0.001
Negative	349 (93.6)	263 (99.2)	86 (79.6)	
Positive	24 (6.4)	2 (0.8)	22 (20.4)	
Hexokinase II				<0.001
Negative	289 (77.5)	229 (86.4)	60 (55.6)	
Positive	84 (22.5)	36 (13.6)	48 (44.4)	
CAIX				<0.001
Negative	348 (93.3)	263 (99.2)	85 (78.7)	
Positive	25 (6.7)	2 (0.8)	23 (21.3)	
MCT4				<0.001
Negative	279 (74.8)	215 (81.1)	64 (59.3)	
Positive	94 (25.2)	50 (18.9)	44 (40.7)	

**Table 3:** Expression of glycolysis-related proteins in follicular neoplasms and Hürthle cell neoplasms.

Parameters	Follicular neoplasm *n* = 265 (%)		Hürthle cell neoplasm *n* = 108 (%)		*p* value
	FA *n* = 153 (%)	FC *n* = 112 (%)	HCA *n* = 81 (%)	HCC *n* = 27 (%)	
GLUT-1					<0.001
Negative	152 (99.3)	111 (99.1)	70 (86.4)	16 (59.3)	
Positive	1 (0.7)	1 (0.9)	11 (13.6)	11 (40.7)	
Hexokinase II					<0.001
Negative	140 (91.5)	89 (79.5)	52 (64.2)	8 (29.6)	
Positive	13 (8.5)	23 (20.5)	29 (35.8)	19 (70.4)	
CAIX					<0.001
Negative	153 (100.0)	110 (98.2)	66 (81.5)	19 (70.4)	
Positive	0 (0.0)	2 (1.8)	15 (18.5)	8 (29.6)	
MCT4 (T)					<0.001
Negative	132 (86.3)	83 (74.1)	49 (60.5)	15 (55.6)	
Positive	21 (13.7)	29 (25.9)	32 (39.5)	12 (44.4)	

FA: follicular adenoma; FC: follicular carcinoma; HCA: Hürthle cell adenoma; HCC: Hürthle cell carcinoma.

**Table 4:** Number of positive markers for glycolysis in follicular neoplasms and Hürthle cell neoplasms.

Parameter	Total *N* = 373 (%)	FA *n* = 153 (%)	FC *n* = 112 (%)	HCA *n* = 84 (%)	HCC *n* = 27 (%)	*p* value
Number of positive markers for glycolysis						<0.001
0	215 (57.6)	120 (78.4)	67 (59.8)	26 (32.1)	2 (7.4)	
1	111 (29.8)	33 (21.6)	36 (32.1)	33 (40.7)	9 (33.3)	
2	28 (7.5)	0 (0.0)	8 (7.1)	12 (14.8)	8 (29.6)	
3	18 (4.8)	0 (0.0)	1 (0.9)	10 (12.3)	7 (25.9)	
4	1 (0.3)	0 (0.0)	0 (0.0)	0 (0.0)	1 (3.7)	

FA: follicular adenoma; FC: follicular carcinoma; HCA: Hürthle cell adenoma; HCC: Hürthle cell carcinoma.

**Table 5:** Correlation between the expression of glycolysis-related proteins and Ki-67 labeling index in follicular carcinomas and Hürthle cell carcinomas.

Parameter	Follicular carcinoma (*n* = 112)		Hürthle cell carcinoma (*n* = 27)	
	Correlation coefficient	*p* value	Correlation coefficient	*p* value
GLUT1	−0.001	0.992	0.419	0.029
Hexokinase II	0.001	0.989	0.076	0.702
CAIX	−0.059	0.531	0.245	0.217
MCT4	0.187	0.048	0.277	0.162

**Table 6:** Cox-proportional hazard analysis for disease-free survival in follicular carcinomas and Hürthle cell carcinomas.

Parameter	Univariate analysis			Multivariate analysis		
	Odd ratio	95% CI	*p* value	Odd ratio	95% CI	*p* value
Age ≥ 45 (years)	3.656	0.789–16.934	0.097			
Female sex	0.208	0.063–0.683	0.009	0.201	0.061–0.660	0.008
Tumor size >2.0 (cm)	5.363	0.686–41.910	0.109			
Capsular invasion	1.320	0.169–10.316	0.790			
Vascular invasion	3.204	0.938–10.946	0.063			
Extrathyroidal extension	4.753	1.447–15.612	0.010	4.937	1.503–16.208	0.008
Lymph node metastasis	n/a	n/a	n/a			
GLUT-1 positivity	n/a	n/a	n/a			
Hexokinase II positivity	0.523	0.113–2.422	0.407			
CAIX positivity	n/a	n/a	n/a			
MCT4 (T) positivity	0.264	0.033–2.066	0.204			
Glycolysis type^∗^	n/a	n/a	n/a			

n/a: not applicable. ^∗^Glycolysis type was defined as those positive for two or more of the glycolysis related proteins.

**Table 7:** Cox-proportional hazard analysis for overall survival in follicular carcinomas and Hürthle cell carcinomas.

Parameter	Univariate analysis			Multivariate analysis		
	Odd ratio	95% CI	*p* value	Odd ratio	95% CI	*p* value
Age ≥ 45 (years)	0.017	0.001–23.502	0.270			
Female sex	0.326	0.053–1.994	0.225			
Tumor size >2.0 (cm)	2.151	0.240–19.242	0.493			
Capsular invasion	0.546	0.060–4.899	0.589			
Vascular invasion	2.530	0.421–15.201	0.310			
Extrathyroidal extension	3.903	0.651–23.401	0.136			
Lymph node metastasis	15.742	1.723–143.770	0.014	15.742	1.723–143.770	0.014
GLUT-1 positivity	n/a	n/a	n/a			
Hexokinase II positivity	n/a	n/a	n/a			
CAIX positivity	n/a	n/a	n/a			
MCT4 (T) positivity	0.790	0.087–7.225	0.838			
Glycolysis type^∗^	n/a	n/a	n/a			

n/a: not applicable. ^∗^Glycolysis type was defined as those positive for two or more of the glycolysis related proteins.
